# Chitosanase Production from the Liquid Fermentation of Squid Pens Waste by *Paenibacillus elgii*

**DOI:** 10.3390/polym15183724

**Published:** 2023-09-11

**Authors:** Chien Thang Doan, Thi Ngoc Tran, Thi Phuong Hanh Tran, Thi Thanh Nguyen, Huu Kien Nguyen, Thi Kim Thi Tran, Bich Thuy Vu, Thi Huyen Trang Trinh, Anh Dzung Nguyen, San-Lang Wang

**Affiliations:** 1 Faculty of Natural Science and Technology, Tay Nguyen University, Buon Ma Thuot 630000, Vietnam; dcthang@ttn.edu.vn (C.T.D.); ttngoc@ttn.edu.vn (T.N.T.); ttphanh@ttn.edu.vn (T.P.H.T.); ntthanh@ttn.edu.vn (T.T.N.); nhkien@ttn.edu.vn (H.K.N.); ttkthi@ttn.edu.vn (T.K.T.T.); vbthuy@ttn.edu.vn (B.T.V.); tthtrang@ttn.edu.vn (T.H.T.T.); 2 Institute of Biotechnology and Environment, Tay Nguyen University, Buon Ma Thuot 630000, Vietnam; nadzung@ttn.edu.vn; 3 Department of Chemistry, Tamkang University, New Taipei City 25137, Taiwan; 4 Life Science Development Center, Tamkang University, New Taipei City 25137, Taiwan

**Keywords:** chitinous fishery wastes, chitosan, chitosanase, chitooligosaccharide, *Paenibacillus elgii*, squid pens

## Abstract

Chitosanases play a significant part in the hydrolysis of chitosan to form chitooligosaccharides (COS) that possess diverse biological activities. This study aimed to enhance the productivity of *Paenibacillus elgii* TKU051 chitosanase by fermentation from chitinous fishery wastes. The ideal parameters for achieving maximum chitosanase activity were determined: a squid pens powder amount of 5.278% (*w*/*v*), an initial pH value of 8.93, an incubation temperature of 38 °C, and an incubation duration of 5.73 days. The resulting chitosanase activity of the culture medium was 2.023 U/mL. A chitosanase with a molecular weight of 25 kDa was isolated from the culture medium of *P. elgii* TKU051 and was biochemically characterized. Liquid chromatography with tandem mass spectrometry analysis revealed that *P. elgii* TKU051 chitosanase exhibited a maximum amino acid identity of 43% with a chitosanase of *Bacillus circulans* belonging to the glycoside hydrolase (GH) family 46. *P. elgii* TKU051 chitosanase demonstrated optimal activity at pH 5.5 while displaying remarkable stability within the pH range of 5.0 to 9.0. The enzyme displayed maximum efficiency at 60 °C and demonstrated considerable stability at temperatures ≤40 °C. The presence of Mn^2+^ positively affected the activity of the enzyme, while the presence of Cu^2+^ had a negative effect. Thin-layer chromatography analysis demonstrated that *P. elgii* TKU051 chitosanase exhibited an endo-type cleavage pattern and hydrolyzed chitosan with 98% degree of deacetylation to yield (GlcN)_2_ and (GlcN)_3_. The enzymatic properties of *P. elgii* TKU051 chitosanase render it a promising candidate for application in the production of COS.

## 1. Introduction

Chitosan is a polysaccharide comprising glucosamine and N-acetylglucosamine units linked together by β-1,4-glycosidic bonds [1]. This versatile compound is a promising non-toxic polymer with remarkable properties such as biocompatibility, antibacterial activity, biodegradability, film-forming ability, and others [2], making it useful for a wide range of applications, including food [3], cosmetics [4], biomedicine [5], agriculture [6], and environmental protection [7]. However, due to its poor solubility at neutral pH values, chitosan has limited usage in the food and medicine industry [8]. Recently, researchers have turned their attention to chitooligosaccharides (COS, the hydrolysis product of chitosan) because of their various biological activities, such as antioxidant, antidiabetic, antifungal, anti-inflammatory, antimicrobial, antitumor, and immuno-enhancing properties [8,9,10,11]. As a result, COS is extensively applied in the domains of agriculture, food, biomedicine, and other related areas [12]. COS can be obtained through chemical, physical, and enzymatic degradation techniques [8,13]. Among these, the enzymatic approach offers several advantages, such as mild reaction conditions, high yields, good selectivity, controllability, reproducibility, and environment friendliness [13]. Chitinase and chitosanase enzymes have high substrate specificity to chitosan, leading to their application in the production of COS.

Chitosanases (EC 3.2.1.132) are a class of enzymes that hydrolyze endo-β-1,4-glycosidic linkages in chitosan, forming COS with different degrees of polymerization (DP) [14]. They are widely distributed in bacteria [15,16], fungi [17], viruses [18], and some plant tissues [19,20]. Chitosanases are classified into six glycoside hydrolase (GH) families: GH 5, GH 7, GH 8, GH 46, GH 75, and GH 80 [21]. Among them, GH 46, GH 75, and GH 80 are specific to chitosan hydrolysis; GH 46 chitosanases have been characterized most extensively compared with other chitosanases. The GH 46 chitosanases have a strong catalytic ability and are found mainly in bacteria [16]. GH 46 chitosanases are endo-enzymes yielding COS (DP ≥ 2) [22]; this property could make them a potentially useful biotechnological tool for the green production of COS. Nevertheless, the high cost of enzyme production hinders the adoption of this method. This limitation can be overcome by lowering the enzyme production cost and potentially reducing the overall cost of COS production [8,23].

The cultivation parameters significantly impact the chitosanase productivity of microbial strains [17], with the cost of culture medium accounting for approximately 30–40% of the total enzyme production cost [23]. Most microbial strains can be induced to synthesize chitosanase in media containing colloidal chitin or chitosan as the primary carbon source [17]. However, the overall production cost may increase due to the cost and preparation process of colloidal chitin or chitosan, thus hindering the large-scale production of chitosanase. Hence, it is crucial to search for affordable substrates for the enzyme production and optimize the cultivation conditions to significantly reduce the cost [24]. Fortunately, chitin and chitosan can be produced from chitinous fishery wastes through deproteinization and demineralization processes [25,26]. This means that chitinous fishery wastes, such as shrimp heads, crab shells, and squid pens [27], can be used directly as a nutrient source for microbial fermentation to produce chitinolytic enzymes [9,28]. One significant advantage of using fishery wastes is that they can serve as both carbon and nitrogen (C/N) sources and as enzyme inducers [27].

The genus *Paenibacillus* comprises endospore-forming and facultative anaerobic bacteria, formerly categorized as constituents of the *Bacillus* genus. Nevertheless, subsequent to a comprehensive comparative analysis of 16S ribosomal RNA sequences, the aforementioned bacteria were reclassified as a unique genus in 1993 [29]. Several species in the genus of *Paenibacillus* have been reported to secrete different chitosanases [8,9,30,31,32,33,34]. Kim et al. (2004) introduced *Paenibacillus elgii* as a novel species of the genus *Paenibacillus* [35]. *P. elgii*, akin to many other *Paenibacillus* species, can generate the chitinolytic enzyme [31]. Nonetheless, the production, characterization, and application of *P. elgii* chitosanase for COS preparation remain yet to be explored. In this study, *P. elgii* TKU051, a strain originally isolated from the soil of Tamkang University [26], was optimized for its chitosanase productivity in a shake-flask culture condition using chitinous fishery wastes as the unique C/N source. The study aimed to purify the *P. elgii* TKU051 chitosanase and characterize its biochemical properties.

## 2. Materials and Methods

### 2.1. Materials

*P. elgii* TKU051 was the same strain as was used in our previous work [8]. Macro-Prep CM and Macro-Prep High S were procured from Bio-Rad (Hercules, CA, USA). Chitosan with ≥75% degree of deacetylation (DDA) and Sephacryl S-200 gel were all obtained from Sigma Co. (St. Louis, MO, USA). High DDA chitosan (98%) was provided by the Microorganisms and Biochemistry Laboratory, Department of Chemistry, Tamkang University, New Taipei, Taiwan. The shrimp heads and shells, crab shells, and squid pens were obtained from Shin-Ma Frozen Food Co. (Su-Ao city, Taiwan). Other chemicals were of the highest purity obtainable.

### 2.2. Chitosanase Assay

The chitosanase activity was determined following a modified version of a previously established method [8]. The chitosan hydrolysis reaction was conducted at pH 5.5, combining equal volumes of the substrate (0.1 mL of 1% *w*/*v* chitosan) and the sample (0.1 mL), with incubation at 60 °C for 30 min. Subsequently, the reaction mixture was mixed with 1.5 mL of the 3,5-dinitrosalicylic acid reagent and heated at 100 °C for 10 min. The absorbance of the developed color was measured at 515 nm on a spectrophotometer to determine the quantity of reducing sugar generated. Chitosanase activity was defined as the enzyme amount required to produce 1 µmol of reducing sugar per minute.

### 2.3. Production Condition Screening

To optimize the production conditions, a systematic screening approach was employed. Various factors potentially influencing chitosanase productivity were investigated using the one-factor-at-a-time (OFAT) method. These factors included the C/N source (crab shells powder, CSP; shrimp shells powder, SSP; shrimp heads powder, SHP; squid pens powder, SPP; colloidal chitin, CC), SPP concentration (0.5% to 6%), pH (5.6 to 9.6), temperature (28 °C to 40 °C), and incubation time (0 to 8 days). The initial conditions for the experiments were set as follows: 1% concentration for each C/N source, 0.1% KH_2_PO_4_, 0.05% MgSO_4_, pH 7.6, and a temperature of 37 °C. One at a time, each factor was varied, while the other factors were kept constant. The condition that resulted in the highest chitosanase activity was selected for further experimentation and optimization.

### 2.4. Production Optimization

Following the preliminary results from the OFAT experiments, the Box-Behnken design of response surface methodology (RSM) was employed to optimize the response of four independent factors: SPP concentration (X_1_), initial pH (X_2_), incubation temperature (X_3_), and incubation time (X_4_). The optimization was conducted using the R-software (2021.09.1+372 version), and a total of 27 runs were performed for the optimization. Each factor was tested at three levels: low, medium, and high, represented by the coded values of −1, 0, and +1, respectively. The resultant data were analyzed using R-software to determine the optimal conditions for the chitosanase production. The following second-order polynomial equation was the effect of four factors:

Y = *β*_0_ + *β*_1_X_1_ + *β*_2_X_2_ + *β*_3_X_3_ + *β*_4_X_4_ + *β*_12_X_1_ × X_2_ + *β*_13_X_1_ × X_3_ + *β*_14_X_1_ × X_4_ + *β*_23_X_2_ × X_3_ + *β*_24_X_2_ × X_4_ + *β*_34_X_3_ × X_4_ + *β*_11_X_1_^2^ + *β*_22_X_2_^2^ + *β*_33_X_3_^2^ + *β*_44_X_4_^2^ (1)

where Y is the predicted response (U/mL); *β*_0_ is the intercept; *β*_1_, *β*_2_, *β*_3_, and *β*_4_ are linear coefficients; *β*_12_, *β*_13_, *β*_14_, *β*_23_, *β*_24_, and *β*_34_ are interactive coefficients; and *β*_11_, *β*_22_, *β*_33_, and *β*_44_ are quadratic coefficients.

### 2.5. Chitosanase Purification

To purify the chitosanase, the culture supernatant (0.4 L) containing the enzyme was precipitated with (NH_4_)_2_SO_4_, and the resulting precipitate was re-dissolved in acetate buffer (50 mM, pH 5.5). To remove any residual (NH_4_)_2_SO_4_, the crude enzyme solution was dialyzed against acetate buffer (50 mM, pH 5.5) for 1 day. Next, the crude enzyme was loaded onto a high S column pre-equilibrated with acetate buffer (50 mM, pH 5.5). The chitosanase bound to the column was eluted using a NaCl gradient (0–0.5 mM). The active fraction containing the enzyme was collected, dialyzed against acetate buffer (50 mM, pH 5.5), and further purified using a CM column (0–0.5 mM). Chitosanase was eluted from the CM column by applying a NaCl gradient. Subsequently, the active fraction was concentrated by the freeze-drying method and loaded onto a Sephacryl S-200 column for final purification. The molecular weight and purity of the purified enzyme were determined by sodium dodecyl sulfate–polyacrylamide gel electrophoresis (SDS-PAGE) [8]. The SDS-PAGE gel was stained by Protein Assay Dye Reagent from BioRad (Hercules, CA, USA). The chitinolytic activity of the purified enzyme was confirmed through native-PAGE containing 0.01% chitosan. In this assay, the chitosanase was loaded onto an acrylamide gel containing 0.01% chitosan and electrophoresed at 140 V and 4 °C. The gel was subsequently incubated overnight at 37 °C in acetate buffer (50 mM, pH 5.5) and finally stained with Congo red (0.1%). The gel was destained with 1 M NaCl; the chitosanase activity band appeared distinct and clear.

### 2.6. Mass Spectrometry and Protein Identification

Briefly, the target band on the SDS-PAGE gel was obtained and subjected to in-gel digestion with trypsin. Following that, the identity of the protein was determined by liquid chromatography-tandem mass spectrometry (LC-MS/MS) analysis performed by Mission Biotech Company (Taipei, Taiwan). The resulting fragment spectra were then searched against the SwissProt database using the MASCOT search engine.

### 2.7. Characterization of Paenibacillus elgii TKU051 Chitosanase

The optimum temperature for the activity of *P. elgii* TKU051 chitosanase was determined by incubating the enzyme with 98% DDA chitosan solution (1%, *w*/*v*) at 30–80 °C in acetate buffer (50 mM, pH 5.5). The thermostability of *P. elgii* TKU051 chitosanase was determined by measuring the residual activity after incubating the enzyme at different temperatures (30–80 °C) for 60 min. The optimum pH for *P. elgii* TKU051 chitosanase activity was determined in the range of pH 3.0 to 10.0 (glycine HCl buffer (pH 3 and 3.5), acetate buffer (pH 4, 5 and 5.5), phosphate (pH 6, 7, and 8), and Tris-HCl buffer (pH 9, 10, and 11)). The residual chitosanase activity was also measured after incubating the enzyme in the above-mentioned buffers at pH 3.0–10.0 at 20 °C for 60 min. The impacts of chemicals (FeCl_2_, CaCl_2_, CuCl_2_, MgCl_2_, MnCl_2_, ZnCl_2_, EDTA (ethylenediaminetetraacetic acid), 2-ME (2-mercaptoethanol), Tween 20, Tween 40, Triton X-100, and SDS (Sodium dodecyl sulfate)) on the enzyme activity were determined by adding these chemicals into the reaction system at a 5 mM final concentration, except for Tween 20, Tween 40, Triton X-100, and SDS, which were added at 1% final concentration; the control for the assay was the reaction mixture with no added chemicals. Substrate specificity of *P. elgii* TKU051 chitosanase was evaluated at 60 °C for 30 min in 50 mM acetate buffer (pH 5.5) using different polysaccharide substrates (1% *w*/*v*), including 98% and 75% DDA chitosan solution, 98% and 75% DDA chitosan powder, colloidal chitin, chitin powder, cellulose, β-1,3 glucan, pectin, starch, and dextran.

### 2.8. Thin-Layer Chromatography (TLC) Analysis of Hydrolysis Products

The hydrolysis products of chitosan catalyzed by *P. elgii* TKU051 chitosanase were analyzed according to protocols mentioned in previous works [8]. The hydrolysis solutions of 98% DDA chitosan with *P. elgii* TKU051 chitosanase at 0 h, 1 h, 2 h, 3 h, 4 h, and 5 h were analyzed by the TLC method. The mobile phase was a mixture of propanol/ammonia solution/water (70/10/20, *v*/*v*/*v*). The hydrolysis products were visualized by spraying the TLC plate with 10% H_2_SO_4_ in ethanol and heating it at 180 °C.

## 3. Results and Discussion

### 3.1. Screening of Carbon/Nitrogen Source and Culture Factors for the Chitosanase Productivity of Paenibacillus elgii TKU051

**Figure 1:**
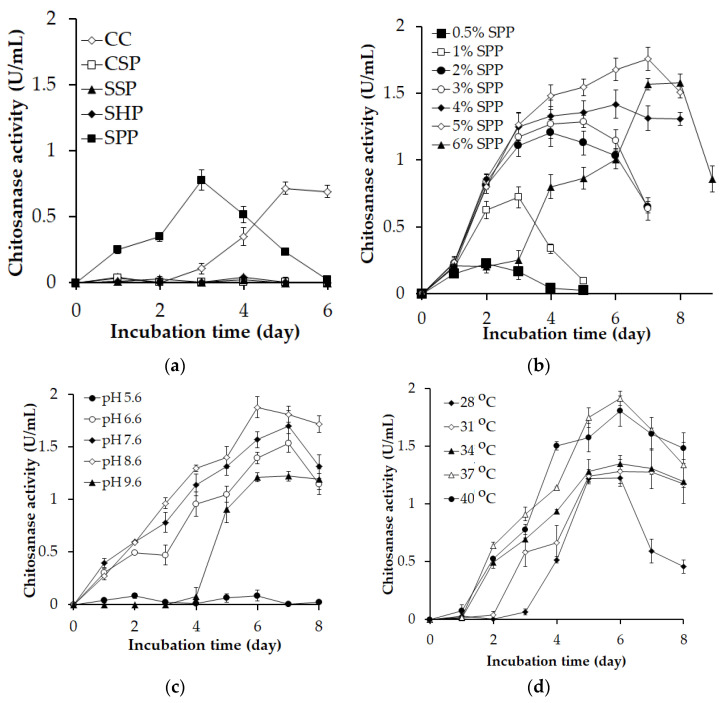
Screening of culture factors for the chitosanase production of *Paenibacillus elgii* TKU051. (**a**), Kind of C/N source; (**b**), amount of SPP; (**c**), initial pH of the medium; (**d**), incubation temperature. CC, colloidal chitin; C/N, carbon/ nitrogen; SPP, squid pen powder; SHP, shrimp head powder; SSP, shrimp shell powder. The error bars represent the standard deviation (SD) of the means of three replicates.

Several low-cost and abundantly available chitin-rich fishery wastes (squid pens, crab shells, shrimp heads, and shrimp shells) were screened for their suitability for chitosanase production (Figure 1a). Among the materials, SPP was the best C/N source for chitosanase production (yielding 0.779 U/mL on the third day), followed by CC (0.718 U/mL on the fifth day). CSP, SSP, and SHP did not appear to be appropriate C/N sources for producing chitosanase, as the enzyme productivity of *P. elgii* TKU051 on the mediums containing these C/N sources was insignificant. Likewise, SPP has been a suitable substrate for chitosanase production by *Paenibacillus* sp. TKU042 [33], *Paenibacillus macerans* TKU029 [36], and *Paenibacillus* sp. TKU047 [8]. However, *Paenibacillus mucilaginosus* TKU032 exhibited the highest chitosanase productivity when SHP was the C/N source [9]. CC is typically considered the optimal substrate for the synthesis of chitinolytic enzymes by various microbial strains [17,37,38,39,40,41]; however, CC is a chitin form produced through chemical treatment, potentially resulting in high production costs and environmental pollution [38]. These drawbacks limit the utilization of CC for large-scale production of chitinolytic enzymes. In this study, *P. elgii* TKU051 demonstrated greater efficiency in chitosanase production when utilizing SPP instead of CC. Thus, SPP emerges as a viable and effective alternative to CC for chitosanase production by *P. elgii* TKU051. Furthermore, when using different amounts of SPP as substrates for chitosanase production, 5% of SPP gave the best yield of chitosanase (1.757 U/mL on day 7) (Figure 1b).

Next, the effect of pH, temperature, and incubation time on the chitosanase production of *P. elgii* TKU051 was explored. As seen in Figure 1c, the chitosanase activity of the culture supernatants was observed at a broad pH range of 6.6 to 9.6, with maximum productivity at pH 8.6 (1.789 U/mL on day 6). Chitosanase production was significantly dependent on cultivation temperature (Figure 1d) and reached a maximum of 1.916 U/mL at 37 °C after six days of culturing. Based on these results, pH 8.6, 37 °C, and six days of culturing were selected as the optimal conditions for producing chitosanase in subsequent experiments.

At present, squid pens are considered an unappealing by-product of the squid processing sector [42]. Nevertheless, several distinctive features of squid pens suggest their potential suitability as a viable chitin source [43]. For sustainable methodologies, the use of squid pens as a viable C/N source in microbial fermentation processes is a promising avenue for producing high-value-added bioactive materials [44,45,46,47]. In this study, squid pens were potentially promising in the production of chitosanase by *P. elgii* TKU051. Further, the culture conditions were optimized to enhance the production of chitosanase.

### 3.2. Production Optimization of Paenibacillus elgii TKU051 Chitosanase

Important parameters, including the amount of SPP (X_1_), initial pH of the medium (X_2_), incubation temperature (X_3_), and incubation time (X_4_), were optimized by using the RSM based on the Box-Behnken design to achieve the highest production of chitosanase. The experimental design was based on 27 experiments, including three central points (Table 1). The chitosanase activity of the cultural mediums ranged from 0.230 U/mL to 1.966 U/mL under different culture conditions. The effect of four factors was the following second-order polynomial equation:

Y = −102.340 − 1.664X_1_ + 2.406X_2_ + 3.674X_3_ + 9.819X_4_ + 0.312X_1_ × X_2_ + 0.033X_1_ × X_3_ + 0.199X_1_ × X_4_ + 0.080X_2_ × X_3_ − 0.042X_2_ × X_4_ − 0.055X_3_ × X_4_ − 0.332X_1_^2^ − 0.383X_2_^2^ − 0.056X_3_^2^ − 0.733X_4_^2^

The results of regression analysis and analysis of variance are presented in Table 2 and Table 3. The F-statistic of the model was evaluated to be 19.87, implying the significance of the model. The lack-of-fit F-value of 2.007 implies the non-significance of the lack of fit relative to the pure error, further confirming that the model was significant. The value of the determination coefficient was 0.9586, indicating that only 4.14% of total variations could not be explained by the model. The adjusted determination coefficient (adjusted R^2^) of all models was also high (0.9104) and confirmed the significance of the model. The chitosanase productivity was strongly affected by the linear terms of X_3_ and X_4_, the 2-factor interactions terms of X_1_X_2_, X_1_X_4_, and X_2_X_3,_ and the quadratic term (Table 2).

**Figure 2:**
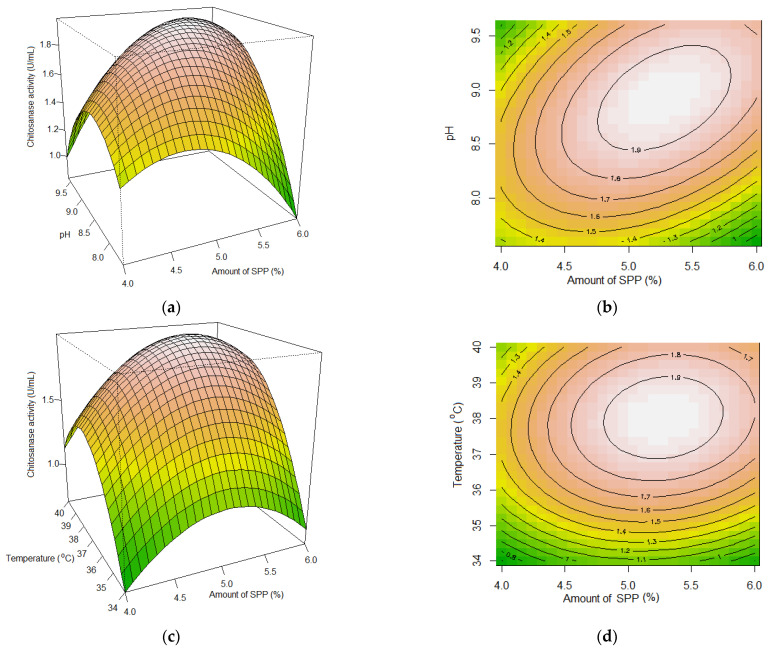

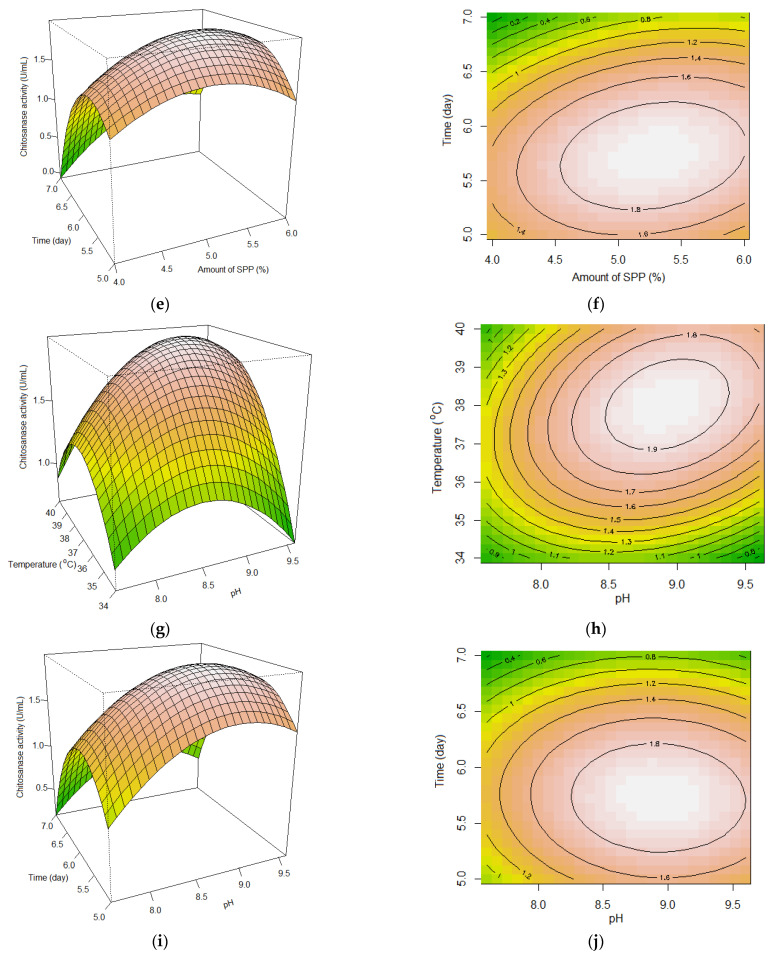

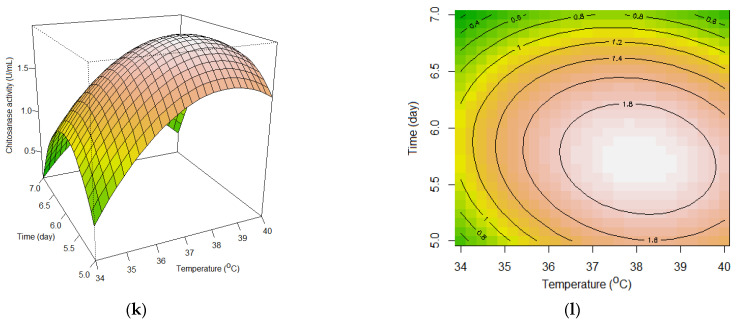
Response surface plots and 2-dimensional contour plots showing the effect of the amount of squid pens powder (SPP), initial pH of medium, temperature, and incubation time on chitosanase productivity of *Paenibacillus elgii* TKU051. (**a**), response surface plot and (**b**), 2-dimensional contour plot showing the effect of amount of SPP and initial pH; (**c**), response surface plot and (**d**), 2-dimensional contour plot showing the effect of amount of SPP and temperature; (**e**), response surface plot and (**f**), 2-dimensional contour plot showing the effect of amount of SPP and incubation time; (**g**), response surface plot and (**h**), 2-dimensional contour plot showing the effect of initial pH and temperature; (**i**), response surface plot and (**j**), 2-dimensional contour plot showing the effect of initial pH and incubation time; (**k**), response surface plot and (**l**), 2-dimensional contour plot showing the effect of temperature and incubation time.

The relationship between the dependent and independent variables and the determination of their optimal levels were explored by generating 2-dimensional (2D) contour plots and 3D response surface plots by plotting one of the responses against any two independent variables while holding the third variable as a constant at its midpoint (0) level (Figure 2). The 3D response surface plots were expected to exhibit a convex shape, with the plot peak indicating the optimal combination of the two factors being tested. Meanwhile, the 2D contour plots displayed an oval shape, indicating a significant interaction between the two variables that were plotted [48]. Thus, the chitosanase activity of the culture medium was susceptible to changes in the tested factors. Specifically, as depicted in Figure 2, the chitosanase activity of the culture medium increased initially and then decreased with the increase in the SPP amount, initial pH of the medium, incubation temperature, and fermentation time.

The maximum predicted chitosanase activity of 1.974 U/mL could be attained by setting the initial pH, incubation temperature, incubation time, and amount of SPP at 8.93, 38 °C, 5.73 days, and 5.278%, respectively. The fermentation was executed in triplicate using the recommended fermentation conditions to validate the optimization findings. *P. elgii* TKU051 demonstrated chitosanase productivity of 2.023 ± 0.072 U/mL under the optimal conditions (Table 4), consistent with the predicted maximum activity and greater than its chitosanase productivity under unoptimized conditions (0.777 ± 0.076 U/mL) and optimization by employing the OFAT method (1.899 ± 0.031 U/mL). Therefore, the production process of chitosanase from *P. elgii* TKU051 using SPP as the sole C/N source could be successfully optimized.

### 3.3. Enzyme Purification and Identification

**Figure 3:**
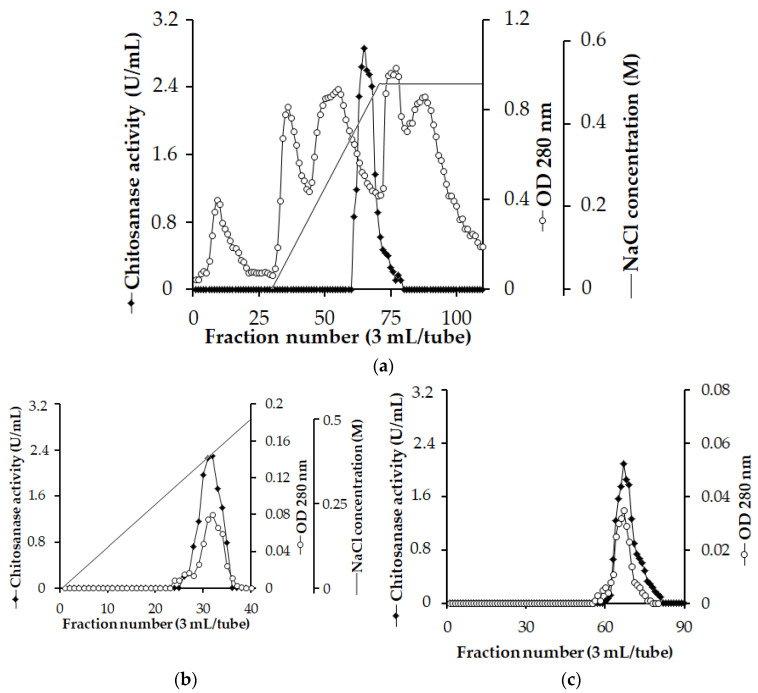
Typical chromatography profiles of the chitosanase purification process. (**a**) High S column, (**b**) CM column, (**c**) Sephacryl S-200 column.

The chitosanase was purified before its characteristics could be investigated further. The crude chitosanase was obtained from the culture supernatant of *P. elgii* TKU051 using the (NH_4_)_2_SO_4_ precipitation method. It was then purified using Macro-Prep High S, CM, and Sephacryl S-200 columns (Figure 3). A unique peak of chitosanase fraction was observed during the purification process. As shown in Table 5, the purified chitosanase demonstrated a 141.081-fold increase in specific activity, and 4% of the total activity could be recovered from the culture supernatant using the purification process.

**Figure 4:**
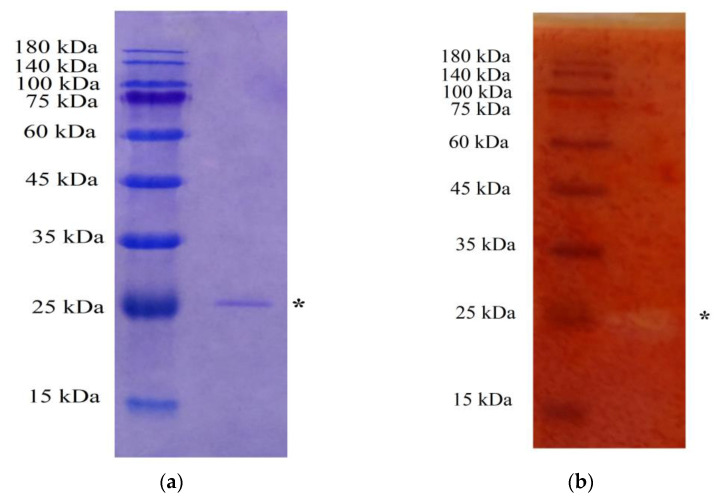
Sodium dodecyl sulfate–polyacrylamide gel electrophoresis (**a**) and zymography (**b**) profiles of the purified chitosanase. *, location of the enzyme.

The molecular weight of the purified chitosanase was determined by SDS-PAGE. As depicted in Figure 4a, the MW of *P. elgii* TKU051 chitosanase was approximately 25 kDa, being smaller than most of the chitosanases from the *Paenibacillus* genus such as chitosanase from *P. mucilaginosus* TKU032 (59 kDa) [9], PbCsn8 from *P. barengoltzii* (60 kDa), chitosanase from *Paenibacillus* sp. TKU042 (70 kDa) [33], chitosanase from *P. macerans* TKU029 (63 kDa) [36], Csn-PD from *Paenibacillus dendritiformis* (31 kDa) [49], Csn1794 from *Paenibacillus* sp. 1794 (40 kDa) [50], and chitosanase from *P. fukuinensis* D2 (81 kDa) [51] but similar to the chitosanase from *Paenibacillus* sp. TKU047 (23 kDa) [8]. The enzyme activity was verified on a polyacrylamide gel containing 0.1% (%v) chitosan. Staining with Congo Red Solution revealed one distance chitinolytic activity band at the 25 kDa position, confirming that the 25 kDa protein band indeed had chitosanase activity (Figure 4b).

The *P. elgii* TKU051 chitosanase band was excised from the SDS-PAGE gel, subjected to trypsin digestion, and analyzed by LC-MS/MS. The MASCOT search result (database: Swissprot, Taxonomy: Firmicutes) revealed that the chitosanase was related to the GH family of 46 chitosanases, with CHIS_BACCI (*Bacillus circulans*) being the closest relative, with 43% amino acid sequence identity (Table 6). In earlier studies, chitosanases from the *Paenibacillus* genus could be classified into the GH 8 family (*P. barengoltzii* [30], *Paenibacillus* sp. 1794 [50], and *P. fukuinensis* [51]) and the GH 46 family (*P. dendritiformis* [49] and *Paenibacillus* sp. 1–18 [34]). The GH 46 chitosanases are usually smaller, with lower molecular weights than those of GH 8 (around 30 kDa) [16], such as *Bacillus* sp. Q1098 30 kDa [52], *Bacillus* sp. DAU101 27 kDa [53], *Bacillus* sp. CK4 32 kDa [54], *Bacillus ehimensis* EAG1 31 kDa [55], *B. circulans* MH-K1 29 kDa [56], *Pseudomonas* sp. A-01 28 kDa [57], *Renibacterium* sp. Y82 30 kDa [15], *Bacillus coagulans* CK108 32 kDa [58], and *Bacillus subtilis* SH21 30.72 kDa [16]. Thus, *P. elgii* TKU051 chitosanase is even smaller than most of the other GH 46 chitosanases. As mentioned above, GH 46 chitosanases are endo-enzymes that produce COS [22]. In one previous study, GH 46 family chitosanases from several *Bacillus* strains revealed higher efficiency than the non-specific enzymes, GH 8 chitosanases, and GH 75 chitosanases [59]. Indeed, several GH 46 chitosanases have been used to generate low molecular weight COS [15,58,59]. Thus, exploring the potential of *P. elgii* TKU051 chitosanase as a biotechnological tool for producing COS is of great interest.

### 3.4. Biochemical Characteristic of Paenibacillus elgii TKU051 Chitosanase

**Figure 5:**
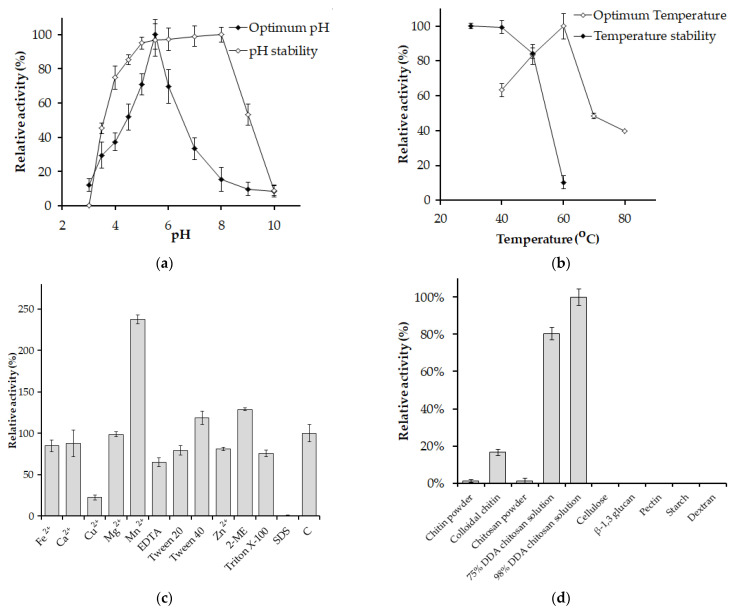
Effect of pH (**a**), temperature (**b**), chemicals (**c**), and substrates (**d**) on the activity of *Paenibacillus elgii* TKU051 chitosanase. EDTA, ethylenediaminetetraacetic acid; 2-ME, 2-mercaptoethanol; SDS, sodium dodecyl sulfate; C, control. DDA, degree of deacetylation. The error bars represent the SD of the means of three replicates.

Figure 5a presents the effect of pH on the activity of *P. elgii* TKU051 chitosanase. The enzyme exhibited its highest activity at pH 5.5 and was stable within the pH range of 5 to 9, retaining over 90% of its residual activity. These findings indicate that the chitosanase from *P. elgii* TKU051 is an acidic enzyme. Similar characteristics have been reported for chitosanases from other *P. barengoltzii* (pH 5.5) [30], *P. mucilaginosus* TKU032 (pH 6) [9], and *Paenibacillus* sp. 1794 (pH 4.8) [50]. In contrast, some chitosanases show optimal activity under the neutral condition (pH 7), such as those from *P. macerans* TKU029 [36], *Paenibacillus* sp. TKU047 [8], and *P. dendritiformis* [49]. Chitosanases demonstrating optimal catalytic activity in acidic environments are advantageous for producing COS because the solubility of chitosan is enhanced at a pH less than 6.0 [59]. Also, with its stability over a broad pH range and optimal acidic pH, *P. elgii* TKU051 chitosanase shows potential for applications in chitosan saccharification processes.

The optimum temperature for the *P. elgii* TKU051 chitosanase was 60 °C (Figure 5b), similar to that of several other reported chitosanases, such as *P. macerans* TKU029 [36] and *Paenibacillus* sp. TKU047 [8], but higher than that of *P. dendritiformis* (45 °C) [49]. Nevertheless, some chitosanases from the *Paenibacillus* genus exhibit higher optimal temperature, such as chitosanases from *P. mucilaginosus* TKU032 (70 °C) [9] and *Paenibacillus* sp. 1794 (80 °C) [50]. The thermal stability of chitosanase from *P. elgii* TKU051 was evaluated by exposing the enzyme solution to different temperatures for 1 h. The results indicate that the chitosanase retained its original activity up to 40 °C, beyond which its activity declined significantly (Figure 5b). At 50 °C, the residual activity of *P. elgii* TKU051 chitosanase dropped only to 84%. At higher temperatures, *P. elgii* TKU051 chitosanase was nearly inactivated. Thus, *P. elgii* TKU051 chitosanase could be considered a thermolabile enzyme. Among GH 46 chitosanases, *P. elgii* TKU051 chitosanase has a higher optimal temperature than that of others, having optimal temperatures in the range of 30–50 °C [13]. The optimal temperature of *P. elgii* TKU051 chitosanase was comparable to some GH 46 chitosanases from *Bacillus* sp. Q1098 (60 °C) [52], *Serratia* sp. QD07 (60 °C) [14], *Streptomyces hygroscopicus* R1 (55 °C) [59], and *Bacillus* sp. CK4 (55 °C) [54].

*P. elgii* TKU051 chitosanase was activated by Mn^2+^, and the residual activity was 238%. Besides, *P. elgii* TKU051 chitosanase was inhibited by Cu^2+^ with residual activity of 23%. Other metal ions had a slight effect on *P. elgii* TKU051 chitosanase. The effect of Mn^2+^ and Cu^2+^ on *P. elgii* TKU051 chitosanase was similar to those reported earlier; the chitosanases from *Paenibacillus* sp. TKU047 [8], *S. hygroscopicus* R1 [59], are activated by Mn^2+^ and inhibited by Cu^2+^. GH46 chitosanases possess metal ion binding sites, where specific metal ions like Mn^2+^ can bind and enhance ’the 3D structural stability and catalytic activity of the chitosanases [60]. Surfactants such as SDS, Triton X-100, and Tween 20 negatively affected *P. elgii* TKU051 chitosanase (residual activity of 0%, 76%, and 79%, respectively), whereas Tween 40 slightly activated *P. elgii* TKU051 chitosanase (residual activity of 119%). *P. elgii* TKU051 chitosanase was significantly affected by EDTA (residual activity of 65%). Interestingly, the activity of *P. elgii* TKU051 chitosanase significantly increased in the presence of 2-ME, to 129% of its initial activity.

*P. elgii* TKU051 chitosanase exhibited the highest relative activity towards 98% DDA chitosan solution (100.00%), followed by 75% DDA chitosan solution (80%), colloidal chitin (17%), chitosan powder (1%), and chitin powder (1%), and exhibited no activity towards other polysaccharide substrates (Figure 5d). Hence, the activity of *P. elgii* TKU051 chitosanase is specific to the GlcN-GlcN linkages. Likewise, a similar phenomenon was observed earlier in chitosanase from *Paenibacillus* sp. TKU047 [8], *P. dendritiformis* [49]. Most GH 46 chitosanases displayed strict substrate specificity towards chitosan with high DDA [49,53]. This result also indicates that the physical form of the substrate significantly affects the activity of chitosanase, with better hydrolytic activity of the enzyme in chitosan solution compared to chitosan powder. Finally, the *K_m_* and *V_max_* of *P. elgii* TKU051 chitosanase were determined at chitosan concentrations ranging from 0.195 mg/mL to 25 mg/mL in sodium acetate buffer (pH 5.5) at 60 °C. The Lineweaver–Burk plot was used to calculate the *K_m_* and *V_max,_* which were at 32.83 mg/mL and 9.200 µM/min, respectively, and the *K_cat_* was 1645 s^−1^. Likewise, the *K_cat_* value of *Bacillus* sp. KCTC 0377BP chitosanase was estimated to be 1600 s^−1^ [61].

### 3.5. Hydrolysis Products

**Figure 6:**
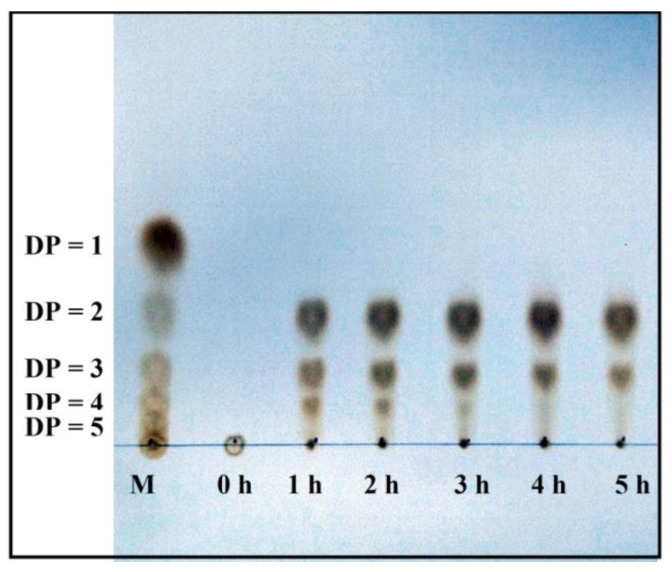
The thin layer chromatography profile of the hydrolysis products of chitosan with 98% degree of deacetylation chitosan solution using *Paenibacillus elgii* TKU051 chitosanase.

The reaction products of *P. elgii* TKU051 chitosanase were examined using a 98% DDA chitosan solution. In the initial stage of the reaction (from 0 h to 1 h), *P. elgii* TKU051 chitosanase rapidly hydrolyzed chitosan, resulting in the formation of (GlcN)_2_, (GlcN)_3_, and (GlcN)_4_ (Figure 6), indicating that *P. elgii* TKU051 chitosanase functions as an endo-chitosanase. From 1 h onward, the levels of (GlcN)_4_ gradually decreased over time, suggesting the hydrolysis of (GlcN)_4_ to generate (GlcN)_2_. Notably, no GlcN product was detected, indicating that *P. elgii* TKU051 chitosanase does not possess exo-β-D-glucosaminidase activity and cannot further degrade (GlcN)_2_ and (GlcN)_3_ into smaller forms. Briefly, *P. elgii* TKU051 chitosanase was determined to be an endo-chitosanase, generating (GlcN)_2_ and (GlcN)_3_ as primary products. Studies have confirmed that certain chitosanases derived from the *Paenibacillus* genus act as endo-chitosanases. This finding is highly consistent with the report by Zhang et al. (2021) [15], who showed that the GH46 family chitosanase CsnY produced (GlcN)_2_ and (GlcN)_3_ as reaction products (Table 7). Most chitosanases from GH 46 display endo-type properties with no activity toward (GlcN)_2_ and (GlcN)_3_. In addition, the majority of GH46 chitosanases seem to favor the generation of COS with lower DP, specifically 2 and 3 [13]. In this study, *P. elgii* TKU051 chitosanase could effectively hydrolyze chitosan to produce COS with a DP of 2–3, and no DP 1 products were observed. Thus, these results underscore the practical application of *P. elgii* TKU051 chitosanase for the production of COS.

## 4. Conclusions

Numerous studies have been done on the production of chitosanase by microbes; nonetheless, there is a dearth of information regarding the statistical optimization of chitosanase production by *P. elgii* utilizing squid pens as a C/N source. Thus, the results of this study may be a novel contribution to this field. In this work, the production optimization of *P. elgii* TKU051 chitosanase using squid pens as the substrate resulted in a 2.6-fold increase (2.023 U/mL) in chitosanase production compared to that in unoptimized conditions. A chitosanase with a molecular weight of 25 kDa was isolated, purified, and biochemically characterized from *P. elgii* TKU051 culture medium. *P. elgii* TKU051 chitosanase was classified under the GH46 family and exhibited optimal activity at 60 °C and pH 5.5. Furthermore, the chitosanase produced by *P. elgii* TKU051 was found to be an endo-type enzyme capable of catalyzing chitosan hydrolysis into low-DP COS. The remarkable characteristics exhibited by the chitosanase of *P. elgii* TKU051 serve as a basis for its use in synthesizing COS.

## Figures and Tables

**Table 1:** Experimental results of the Box-Behnken design.

Run	Coded Levels				Uncoded Levels				Chitosanase Activity (U/mL)
	X_1_	X_2_	X_3_	X_4_	X_1_	X_2_	X_3_	X_4_	
1	−1	0	1	0	4	8.6	40	6	1.140
2	0	0	−1	−1	5	8.6	34	5	0.712
3	0	0	0	0	5	8.6	37	6	1.966
4	0	0	0	0	5	8.6	37	6	1.740
5	0	1	−1	0	5	9.6	34	6	0.826
6	0	1	0	−1	5	9.6	37	5	1.162
7	0	1	0	1	5	9.6	37	7	0.426
8	−1	0	0	−1	4	8.6	37	5	1.300
9	0	0	−1	1	5	8.6	34	7	0.230
10	1	0	0	1	6	8.6	37	7	0.656
11	1	0	0	−1	6	8.6	37	5	1.106
12	0	−1	−1	0	5	7.6	34	6	0.910
13	0	−1	0	1	5	7.6	37	7	0.376
14	0	0	1	−1	5	8.6	40	5	1.426
15	−1	0	0	1	4	8.6	37	7	0.053
16	1	1	0	0	6	9.6	37	6	1.576
17	−1	1	0	0	4	9.6	37	6	0.680
18	1	−1	0	0	6	7.6	37	6	1.063
19	−1	0	−1	0	4	8.6	34	6	0.736
20	−1	−1	0	0	4	7.6	37	6	1.413
21	0	−1	1	0	5	7.6	40	6	0.613
22	0	1	1	0	5	9.6	40	6	1.486
23	0	0	1	1	5	8.6	40	7	0.283
24	1	0	1	0	6	8.6	40	6	1.480
25	0	0	0	0	5	8.6	37	6	1.880
26	1	0	−1	0	6	8.6	34	6	0.683
27	0	−1	0	−1	5	7.6	37	5	0.944

X_1_, amount of squid pens powder (SPP, %); X_2_, initial pH of medium; X_3_, incubation temperature (°C); X_4_, incubation time (day).

**Table 2:** Results of regression analysis of Box-Behnken design.

Term	Coefficient Estimate	Standard Error Coefficient	t Value	Pr (>|t|)	
Constant	−102.340	19.185	−5.335	0.0002	***
X_1_	−1.664	1.421	−1.171	0.2644	
X_2_	2.406	1.614	1.491	0.1618	
X_3_	3.674	0.627	5.858	0.0001	***
X_4_	9.819	1.466	6.696	<0.0001	***
X_1_X_2_	0.312	0.077	4.027	0.0017	**
X_1_X_3_	0.033	0.026	1.270	0.2281	
X_1_X_4_	0.199	0.077	2.576	0.0243	*
X_2_X_3_	0.080	0.026	3.093	0.0093	**
X_2_X_4_	−0.042	0.077	−0.543	0.5971	
X_3_X_4_	−0.055	0.026	−2.137	0.0539	
X_1_^2^	−0.332	0.067	−4.955	0.0003	***
X_2_^2^	−0.383	0.067	−5.722	0.0001	***
X_3_^2^	−0.056	0.007	−7.495	<0.0001	***
X_4_^2^	−0.733	0.067	−10.949	<0.0001	***

R^2^ = 0.9586, Adjusted R^2^ = 0.9104, F-statistic: 19.87 on 14 and 12 DF (degrees of freedom), Signif. codes: 0 ‘***’ 0.001 ‘**’ 0.01 ‘*’ 0.05. X_1_, amount of squid pens powder (SPP, %); X_2_, initial pH of medium; X_3_, incubation temperature (°C); X_4_, incubation time (day).

**Table 3:** Analysis of variance for the fitted quadratic polynomial model.

Source	Degrees of Freedom	Sum of Squares	Mean Square	F Value	Pr (>F)
Fo	4	2.423	0.606	25.314	<0.0001
TWI	6	0.931	0.155	6.483	0.003
PQ	4	3.303	0.826	34.504	<0.0001
Residuals	12	0.287	0.024		
Lack of fit	10	0.261	0.026	2.007	0.378
Pure error	2	0.026	0.013		

**Table 4:** Production comparison of *Paenibacillus elgii* TKU051 chitosanase on different culture conditions.

Factor	Before Optimization	After Optimization	
		By OFAT	By RSM
Amount of SPP (%)	1	5	5.278
Initial pH	7.6	8.6	8.93
Incubation temperature (°C)	37	37	38
Incubation time (day)	3	6	5.73
Chitosanase activity (U/mL)	0.777 ± 0.076	1.899 ± 0.031	2.023 ± 0.072

OFAT, one factor at a time; RSM, response surface methodology.

**Table 5:** A summary of the purification of *Paenibacillus elgii* TKU051 chitosanase.

Step	Total Protein (mg)	Total Activity (U)	Specific Activity (U/mg)	Recovery (%)	Purification (fold)
Cultural supernatant	3541.060	772.933	0.218	100	1.000
(NH_4_)_2_SO_4_ precipitation	642.982	314.496	0.489	41	2.241
High S column	17.938	96.945	5.404	13	24.759
CM column	1.782	44.588	25.019	6	114.619
Sephacryl S-200 column	0.990	30.490	30.795	4	141.081

**Table 6:** Identification of *Paenibacillus elgii* TKU051 chitosanase by liquid chromatography with tandem mass spectrometry analysis.

Matched Peptides Sequence	Identified Protein and Coverage Rate	Strain
^74^LINKPEQDDLNWIKYYGYCEDIEDERGYTIGLFGATTGGSR^114^ ^132^GASNPSADGALKRLGINGKMKGSILEIKDSEK^163^ ^172^LQNDAAWR^179^ ^193^YSVEQAR^199^ ^252^RTLVVDTNKYNKPPNGK^268^ ^273^QWDTLVDMGK^282^ ^287^NVDSEIAQVTDWEMK^301^	Chitosanase CHIS_BACCI (GH46) 43%	*Bacillus circulans*

**Table 7:** Conversion of chitosan to chitooligosaccharides by some endo-chitosanases.

Enzyme Source	Chitosan	DP of Major Product	Detection Method	References
*P. elgii* TKU051	98% DDA	2–3	TLC	This study
*S. hygroscopicus* R1	95% DDA	2–6	TLC	[59]
*Renibacterium* sp. Y82	95% DDA	2–3	TLC and ESI-MS	[15]
*Paenibacillus* sp. TKU047	98% DDA	2–4	TLC	[8]
*P. dendritiformis*	-	2	TLC and ESI-MS	[49]
*Bacillus* sp. Q1098	≥95% DDA	2–3	TLC and ESI-MS	[52]
*P. barengoltzii*	85% DDA	2–3	TLC	[30]
*Bacillus cereus* TKU034	90% DDA	3–9	MALDI-TOF-MS	[62]
*Pseudoalteromonas* sp. SY39	-	2–3	TLC and HPLC	[63]
*Bacillus* sp. DAU101	-	2–6	TLC	[53]
*Bacillus mycoides* TKU038	85% DDA	1–8	HPLC and MALDI-TOF-MS	[64]
*Bacillus thuringiensis*	≥90% DDA	2–5	ESI-Q-TOF and HPAEC-PAD	[65]
*Streptomyces niveus*	94% DDA	2–4	TLC and HPLC	[13]
*Serratia* sp. QD07	≥95% DDA	2–3	TLC	[14]
*Bacillus amyloliquefaciens*		2–3	TLC	[66]

-, Data not available. ESI-MS, electrospray ionization mass spectrometry; MAL-DI-TOF-MS, matrix-assisted laser desorption/ionization time-of-flight mass spectrometry; HPLC, high-performance liquid chromatography; ESI-Q-TOF, electrospray ionization quadrupole time-of-flight mass spectrometry; HPAEC-PAD, high-performance anion exchange chromatography with pulsed amperometric detection.

## Data Availability

Not applicable.
